# Supplementary material for: Danofloxacin Treatment Alters the Diversity and Resistome Profile of Gut Microbiota in Calves
Source: Microorganisms. 2021 Sep 24;9(10):2023. doi: 10.3390/microorganisms9102023 (PMC8538188; doi:10.3390/microorganisms9102023)
Supplement: Supplementary file 1 [file microorganisms-09-02023-s001.zip › microorganisms-1392920-supplementary.pdf]

Table S1. The relative abundance of bacterial phyla of 30 study calves at seven sampling days over four weeks of the study period.

**Phylum**	**Sampling day (relative abundance, %)**						
	**2**	**6**	**14**	**18**	**19**	**22**	**28**
Euryarchaeota (Archaea)	0.91	1.13	0.82	0.48	0.72	0.36	0.73
Actinobacteria	1.33	0.89	0.75	0.84	1.32	0.46	0.50
Bacteroidetes	36.13	38.43	28.37	28.94	27.81	32.29	28.11
Cyanobacteria	0.93	0.97	1.44	0.95	0.42	0.67	1.25
Elusimicrobia	0.59	0.38	0.21	0.20	0.12	0.46	0.30
Fibrobacteres	0.02	0.03	0.02	0.01	0.01	0.01	0.02
Firmicutes	51.93	49.73	56.87	57.64	60.50	55.14	59.67
Fusobacteria	0.01	0.00	0.00	0.00	0.00	0.00	0.00
Lentisphaerae	0.01	0.08	0.01	0.02	0.01	0.04	0.03
Planctomycetes	0.00	0.01	0.14	0.30	0.14	0.48	0.57
Proteobacteria	3.00	2.26	2.72	2.90	1.15	1.63	2.20
Spirochaetes	1.94	3.07	1.68	1.53	1.07	1.34	2.15
Synergistetes	0.01	0.09	0.02	0.01	0.01	0.01	0.03
Tenericutes	2.18	1.24	2.08	1.67	1.94	1.86	1.93
Verrucomicrobia	1.03	1.68	4.86	4.51	4.78	5.25	2.51

Table S2. Comparisons of the relative abundance of bacterial taxa between control (group A) and treatment (group B—non-BRD-induced and group C—BRD-induced) groups.

		**Relative abundance (%)**			**All groups**	**Pair-wise comparison**		
Phyla	Classes	**A**	**B**	**C**	(*p**)	**A-B** (*p*)	**A-C** (*p*)	**B-C** (*p*)
Euryarchaeota	*Methanobacteria*	0.52	0.82	0.81	0.000	0.001	0.000	0.572
	Thermoplasmata	0.00	0.01	0.01	0.000	0.000	0.005	0.233
Acidobacteria	*Holophagae*	0.00	0.00	0.00	0.363	NA	NA	NA
Actinobacteria	*Actinobacteria*	0.40	0.47	0.13	0.000	0.513	0.000	0.000
	Coriobacteriia	0.49	0.63	0.53	0.371	NA	NA	NA
Bacteroidetes	*Bacteroidia*	31.18	30.89	32.80	0.268	NA	NA	NA
Cyanobacteria	*4C0d-2*	0.83	1.08	0.87	0.496	NA	NA	NA
	Chloroplast	0.00	0.00	0.00	0.189	NA	NA	NA
Elusimicrobia	*Elusimicrobia*	0.27	0.39	0.32	0.842	NA	NA	NA
Fibrobacteres	*Fibrobacteria*	0.02	0.01	0.02	0.041	0.096	0.716	0.057
Firmicutes	*Bacilli*	0.07	0.05	0.05	0.021	0.057	0.029	0.695
	Clostridia	56.85	55.48	53.49	0.063	NA	NA	NA
	Erysipelotrichi	0.43	0.64	0.59	0.002	0.001	0.083	0.145
Planctomycetes	*Planctomycetia*	0.60	0.13	0.04	0.000	0.008	0.008	0.002
Proteobacteria	*Alphaproteobacteria*	0.34	0.51	0.43	0.130	NA	NA	NA
	Betaproteobacteria	0.24	0.36	0.51	0.000	0.011	0.000	0.033
	Deltaproteobacteria	0.85	0.74	0.65	0.130	NA	NA	NA
	Epsilonproteobacteria	0.06	0.30	0.32	0.004	0.003	0.038	0.368
	Gammaproteobacteria	0.47	0.50	0.46	0.275	NA	NA	NA
Spirochaetes	*Spirochaetes*	1.97	2.22	1.34	0.067	NA	NA	NA
Synergistetes	*Synergistia*	0.05	0.02	0.02	0.000	0.000	0.000	0.877
Tenericutes	*Mollicutes*	1.17	1.87	1.57	0.000	0.000	0.009	0.200
	RF3	0.21	0.24	0.36	0.010	0.716	0.016	0.031
	Verruco-5	0.09	0.13	0.05	0.144	NA	NA	NA
Verrucomicrobia	*Verrucomicrobiae*	2.87	2.47	4.61	0.006	0.549	0.032	0.008

*Adjusted p-value, the Kruskal–Wallis test was used to compare the three groups and Dunn test for pair-wise comparisons. NA—not applicable to carry out a pair-wise comparison if the test performed by the Kruskal–Wallis test does not show a significant difference among the groups.

Table S3. The Spearman rank correlation test between *Campylobacter* and other genera in the whole fecal samples (all groups combined). *P*-value was adjusted using the False Discovery Rate (FDR)

**Phylum**	**Order**	**Family**	**Genus**	**R***	**FDR**
Actinobacteria	Actinomycetales	Microbacteriaceae	unclassified	0.45	0.000
Bacteroidetes	Bacteroidales	[Odoribacteraceae]	Odoribacter	-0.22	0.016
	Bacteroidales	[Paraprevotellaceae]	[Prevotella]	-0.20	0.044
	Bacteroidales	[Paraprevotellaceae]	CF231	-0.29	0.001
	Bacteroidales	Unclassified	unclassified	0.27	0.001
	Bacteroidales	Bacteroidaceae	Bacteroides	-0.19	0.047
	Bacteroidales	Porphyromonadaceae	Parabacteroides	-0.19	0.049
	Bacteroidales	Prevotellaceae	Prevotella	-0.27	0.001
	Bacteroidales	S24-7	unclassified	-0.29	0.001
Cyanobacteria	YS2	Unclassified	unclassified	0.22	0.017
Euryarchaeota	E2	[Methanomassiliicoccaceae]	vadinCA11	0.34	0.000
Firmicutes	Clostridiales	Christensenellaceae	unclassified	0.27	0.002
	Clostridiales	Eubacteriaceae	Anaerofustis	0.22	0.017
	Clostridiales	Lachnospiraceae	Blautia	-0.20	0.035
	Clostridiales	Peptococcaceae	unclassified	0.27	0.001
	Clostridiales	Veillonellaceae	Phascolarctobacterium	-0.25	0.003
	SHA-98	Unclassified	unclassified	0.20	0.031
Proteobacteria	Unclassified	Unclassified	unclassified	0.27	0.001
	Desulfovibrionales	Desulfovibrionaceae	Desulfovibrio	-0.22	0.015
	Desulfovibrionales	Desulfovibrionaceae	unclassified	0.47	0.000
	Rhizobiales	Hyphomicrobiaceae	Devosia	0.28	0.001

*R – correlation coefficient

Table S4. The Spearman rank correlation test between *Campylobacter* and other genera in pre- and post-treatment samples (groups B and C combined). *P*-value was adjusted using the False Discovery Rate (FDR).

**Phylum**	**Order**	**Family**	**Genus**	**R***	**FDR**
**Pre-Treatment**					
Actinobacteria	Actinomycetales	Microbacteriaceae	unclassified	0.45	0.002
Bacteroidetes	Bacteroidales	[Paraprevotellaceae]	CF231	-0.35	0.038
	Bacteroidales	Prevotellaceae	Prevotella	-0.33	0.053
Firmicutes	Bacillales	Planococcaceae	Lysinibacillus	0.63	0.000
	Clostridiales	Lachnospiraceae	Anaerostipes	0.42	0.005
	Erysipelotrichales	Erysipelotrichaceae	Bulleidia	0.35	0.035
Proteobacteria	Desulfovibrionales	Desulfovibrionaceae	unclassified	0.39	0.012
Verrucomicrobia	Verrucomicrobiales	Verrucomicrobiaceae	Akkermansia	0.48	0.000
**Post-Treatment**					
Actinobacteria	Coriobacteriales	Coriobacteriaceae	Slackia	0.43	0.024
Bacteroidetes	Bacteroidales	[Odoribacteraceae]	Odoribacter	-0.40	0.046
	Bacteroidales	p-2534-18B5	unclassified	0.45	0.015
Cyanobacteria	YS2	unclassified	unclassified	0.46	0.014
Elusimicrobia	Elusimicrobiales	Elusimicrobiaceae	unclassified	0.53	0.002
Euryarchaeota	E2	[Methanomassiliicoccaceae]	vadinCA11	0.46	0.014
Firmicutes	Clostridiales	Christensenellaceae	unclassified	0.41	0.037
	Clostridiales	Dehalobacteriaceae	Dehalobacterium	0.42	0.032
	Clostridiales	Eubacteriaceae	Anaerofustis	0.39	0.052
	Lactobacillales	Carnobacteriaceae	unclassified	0.40	0.046
Proteobacteria	RF32	unclassified	unclassified	0.47	0.012
	Desulfovibrionales	Desulfovibrionaceae	unclassified	0.57	0.000

*R—correlation coefficient

Table S5. Summary of antimicrobial resistance genes detected in pre-and post-treatment fecal samples along with their respective bacterial reservoirs in calf group B. The number of hits for the respective ARG is indicated in the parentheses. NA (not available) represents undetected bacterial taxon.

Phylum	Lower level	Pre-treatment	Post-treatment
Actinobacteria	bacterium_OL_1	ermB(1*)	tetW(6)
	Isoptericola_variabilis_225	NA**	tetQ(5)
	Bifidobacterium_merycicum_DSM_6492	tetW(2)	NA
	Parascardovia_denticolens_IPLA_20019	tetW(1)	NA
	Streptomyces_coelicolor_A3_2_	ermG(14), tetW(2)	NA
	Mycobacterium_abscessus_subsp._bolletii_str._GO_06	NA	tetW(10)
	Mycobacterium_intracellulare_MOTT_02	NA	ant9(80), tet40(1), tetW(11)
	Olsenella_profusa_F0195	NA	aph2(1), aph3(1), ant9(2), tet40(1), tetW(10)
Arthropoda	Glyphodes pyloalis	tetQ(1)	NA
	Pogonomyrmex_barbatus	NA	ant6(1), tet40(20), tet44(1), tetO(2), tetQ(1), tetW(1)
Bacteroidetes	Alistipes_finegoldii_DSM_17242	NA	cfX(1), tet40(4), tetQ(4), tetW(1), tetX(1)
	Bacteroides_stercoris_ATCC_43183	NA	tet40(2), tetQ(2), tetW(2)
	Flavobacterium_indicum_GPTSA100_9_DSM_17447	NA	tet40(1), tetW(31)
	Prevotella_sp.	tetQ(1)	ant6(3), cfX(1), ermF(1), ermG(1), mefE(1), cfR(2), tet40(7), tetQ(170), tetW(5)
	Prevotella_stercorea_DSM_18206	NA	aph2(1), aph3(1), tetQ(18)
Euryarchaeota	Methanobrevibacter_ruminantium_M1	ant9(1), tetO(1)	No ARGs
	Methanosphaera_stadtmanae_DSM_3091	tetA(2)	NA
Firmicutes	Anaerotruncus_sp._CAG_528	NA	tet32(3), tet40(5), tetO(3)
	Bacillus_cereus_FRI_35	NA	ant6(1), cfR(1), tetW(2)
	Butyrivibrio_sp._AE2032	NA	aph2(2), aph3(2), ant6(4), sat(3), cfR(1)
	Clostridium_aminophilum_DSM_10710	NA	tet40(13), tetW(12)
	Clostridium_sp.	aph2(15), aph3(7), ant6(7), ant9(8), sat(7), ermB(1), tet40(1), tet44(1), tetO(10), tetQ(1), tetW(5)	aph2(9), aph3(9), ant6(13), sat(13), ermG(2), tet40(3), tetL(63), tetW(215)
	Dorea_sp._CAG_317	NA	tet40(19), tetO(3), tetW(10)
	Erysipelotrichaceae_bacterium_5_2_54FAA	NA	tet40(2), tetO(2), tetW(1)
	Ethanoligenens_harbinense_YUAN_3	NA	tetW(32)
	Eubacterium_cellulosolvens_6	tet40(1), tetO(10), tetW(2)	NA
	Eubacterium_nodatum_ATCC_33099	NA	tetW(15)
	Eubacterium_rectale_ATCC_33656	NA	tet40(5), tetO(6), tetW(55)
	Eubacterium_sp.	ant6(4), cfX(1), tet40(3), tet44(2), tetO(3), tetW(5)	ant6(2), tet32(3), tet40(54), tetO(12), tetW(314)
	Eubacterium_ventriosum_ATCC_27560	NA	aph2(9), aph3(9), ant9(4), tet40(4), tetW(1)

	Firmicutes_bacterium_CAG	tet40(4)	aph2(1), aac6(1), ant9(31), tet40(476), tetW(388)
	Lachnospiraceae_bacterium_10_1	NA	tet40(7), tetW(1)
	Lactobacillus_plantarum_4_3	ant6(1), tet40(10), tetO(8)	NA
	Lactobacillus_rhamnosus_ATCC_8530	NA	tetW(1)
	Oscillibacter_ruminantium_GH1	NA	tet40(283), tetW(354)
	Oscillibacter_sp._CAG_241	NA	aph2(1), aac6(1), ant6(1), ant9(1), cfR(1), tet40(1), tetA(1), tetW(55)
	Phascolarctobacterium_succinatutens_YIT_12067	NA	tet40(13), tetO(1), tetQ(1), tetW(5)
	Listeria_monocytogenes_M7	tet32(1), tet40(5), tetO(2)	NA
	Roseburia_intestinalis_L1_82	tetB(1), tetO(10)	NA
	Roseburia_sp.	tetO(2)	NA
	Ruminococcus_sp.	tetO(1)	tet40(119), tetO(3), tetQ(1), tetW(6)
	Ruminococcus_albus_7_DSM_20455	NA	tet40(73), tetO(25), tetW(2)
	Ruminococcus_flavefaciens_ATCC_19208	NA	tet40(1)
	Ruminococcus_lactaris_CC59_002D	NA	ant6(3), ant9(6), cfR(3), tet40(5), tetO(2), tetW(1)
	Staphylococcus_warneri_SG1	ant6(1), tetW(1)	NA
	Streptococcus_agalactiae_GD201008_001	tet40(1), tetW(16)	NA
	Sharpea_azabuensis_DSM_18934	aph2(6), aph3(6), ant6(6), ant9(16), sat(6), cfR(12), tetA(1), tetW(3)	NA
	Sulfobacillus_acidophilus_TPY	NA	ant6(1), sat(1), tetW(6)
Proteobacteria	Azospirillum_brasilense_Sp245	NA	tet40(1), tetW(17)
	Burkholderia_cenocepacia	NA	aph2(1), aph3(1), tet40(16), tetW(14)
	Bradyrhizobium_japonicum_USDA_6	tet32(1), tet40(6), tetO(1), tetW(2)	NA
	Campylobacter_concisus_UNSWCS	aph2(2), aph3(1), ant6(1), ant9(1), sat(1), tetO(2)	NA
	Marinobacter_adhaerens_HP15	tetO(1)	NA
	Geobacter_sulfurreducens_PCA	NA	aph2(1), aac6(1), ant6(1), ant9(1), cfR(1), tet40(8), tetQ(256), tetW(4)
	Hyphomicrobium_nitrativorans_NL23	NA	tet40(3), tetL(1), tetW(18)
	Klebsiella_oxytoca_KCTC_1686	NA	tet40(1)
	Pseudoxanthomonas_sp._GW2	NA	tet40(1), tetW(433)
Spirochaetes	Brachyspira_pilosicoli_WesB	NA	ant6(1), sat(1), tet40(2), tet44(1), tetW(491)
Tenericutes	Mycoplasma_sp._CAG_877	NA	No ARGs
	Mycoplasma_dispar	tet40(1)	NA
Verrucomicrobia	Akkermansia_muciniphila_ATCC_BAA_835	tet40(1)	ant6(1), cfR(1), tet40(2), tetW(1)

* Number of hits; **NA—not available, this bacterial taxon was not detected. .
